# Enzyme-Linked Immunosorbent Spot Assay for the Detection of Wilms’ Tumor 1-Specific T Cells Induced by Dendritic Cell Vaccination

**DOI:** 10.3390/biomedicines3040304

**Published:** 2015-12-04

**Authors:** Yumiko Higuchi, Terutsugu Koya, Miki Yuzawa, Naoko Yamaoka, Yumiko Mizuno, Kiyoshi Yoshizawa, Koichi Hirabayashi, Takashi Kobayashi, Kenji Sano, Shigetaka Shimodaira

**Affiliations:** 1 Center for Advanced Cell Therapy, Shinshu University Hospital, Matsumoto 390-8621, Japan; E-Mails: sasa0922@shinshu-u.ac.jp (Y.H.); koya@shinshu-u.ac.jp (T.K.); m1k1yz@shinshu-u.ac.jp (M.Y.); naoko4152@shinshu-u.ac.jp (N.Y.); purimeron@shinshu-u.ac.jp (Y.M.); k.yoshizawa0228@gmail.com (K.Y.); kohira@shinshu-u.ac.jp (K.H.); 2 Department of Health and Medical Sciences, Shinshu University Graduate School of Medicine, Matsumoto 390-8621, Japan; 3 Shinshu Cancer Center, Shinshu University Hospital, Matsumoto 390-8621, Japan; E-mail: takob@shinshu-u.ac.jp; 4 Department of Laboratory Medicine, Shinshu University Hospital, Matsumoto 390-8621, Japan; E-mail: kenjisa@shinshu-u.ac.jp

**Keywords:** enzyme-linked immunosorbent spot assay, tetramer analysis, dendritic cells, antigen-specific cytotoxic T cells, Wilms’ tumor 1

## Abstract

Background: Despite recent advances in cancer immunotherapy and the development of various assays for T cell assessment, a lack of universal standards within immune monitoring remains. The objective of this study was to evaluate the enzyme-linked immunosorbent spot (ELISpot) assay in comparison with major histocompatibility complex-tetramer analysis in the context of dendritic cell (DC)-based cancer immunotherapy. Methods: The ELISpot assay was performed on peripheral blood mononuclear cells to assess reproducibility, daily precision, and linearity using HLA-A*24:02-restricted Cytomegalovirus peptide. Wilms’ tumor 1 (WT1) antigen-specific cytotoxic T cells were then evaluated by both the ELISpot assay and WT1 tetramer analysis in peripheral blood from 46 cancer patients who received DC vaccinations pulsed with human leukocyte antigen (HLA)-A*24:02-restricted modified WT1 peptides. Results: The ELISpot assay was proven to have reproducibility (coefficient of variation (CV) ranged from 7.4% to 16.3%), daily precision (CV ranged from 5.0% to 17.3%), and linearity (*r* = 0.96–0.98). WT1-specific immune responses were detected by the ELISpot assay in 34 out of 46 patients (73.9%) post-vaccination. A Spearman’s rank-correlation coefficient of 0.82 between the ELISpot assay and WT1 tetramer analysis was obtained. Conclusion: This is the first report of a comparison of an ELISpot assay and tetramer analysis in the context of dendritic cell (DC)-based cancer immunotherapy. The ELISpot assay has reproducibility, linearity, and excellent correlation with the WT1 tetramer analysis. These findings suggest that the validated ELISpot assay is useful to monitor the acquired immunity by DC vaccination targeting WT1.

## 1. Introduction

Over the last two decades, cancer immunotherapy by dendritic cell (DC) vaccination has become viable and a number of clinical trials have been conducted [1,2,3,4]. DCs, antigen-presenting cells of the mammalian immune system, can induce antigen specific cytotoxic T lymphocytes (CTLs) through interactions of the major histocompatibility complexes (MHCs) [1].

As Wilms’ tumor 1 (WT1) molecules are expressed in various types of solid tumors, DC vaccination targeting this molecule for cancer patients has priority as an immunotherapy [2,3,4,5,6,7].

Notably, the monitoring of immune response has been explored to detect cancer associated antigen-specific cytotoxic T lymphocytes (CTLs) exhibiting antitumor effects. Various types of methods are used for assessing the variables of immunity, such as MHC tetramer analysis, intracellular cytokine assay, and interferon gamma (IFN-γ) real time polymerase chain reaction (RT-PCR) [8,9,10].

The enzyme-linked immunosorbent spot (ELISpot) assay is an additional method to analyze functional release of IFN-γ from CTLs upon exposure to cancer-associated antigens.

In our facilities, the tetramer analysis has been routinely performed to evaluate the immunological effect of DC vaccination. Although this method could detect frequencies of epitope-specific CTLs, it does not necessarily reflect the function of CTLs. Therefore, we evaluated WT1 antigen-specific CTLs (WT1-CTLs) using the IFN-γ ELISpot assay in comparison with the MHC tetramer analysis as a validation method of DC-based cancer immunotherapy. This is the first study to compare these assays in the context of DC vaccination therapy.

## 2. Materials and Methods

### 2.1. Study Design

The primary objective of the current study was to validate the ELISpot assay as a tool for detection of CTLs. The secondary objective of the current study was to evaluate WT1-CTLs by the ELISpot assay in comparison with the tetramer analysis in patients who have received DC-based cancer immunotherapy.

### 2.2. Patient Population

Forty-six HLA-A*24:02 positive patients with carcinomas, including 11 lung, 6 breast, 5 stomach, 14 colorectal, and 10 pancreas cancer patients were enrolled for WT1 peptide (CYTWNQMNL, residue 235–243) (NeoMPS Inc., San Diego, CA, USA) -pulsed dendritic cell therapy after informed consent. The DC vaccination study was conducted at the Shinshu University Hospital and was approved by the Ethics Committee of the Shinshu University School of Medicine (approval number 1199, 2 December 2008; 2704, 8 April 2014). 

### 2.3. Isolation of Peripheral Blood Mononuclear Cells (PBMCs)

Peripheral blood samples were obtained from patients using BD Vacutainer™ Blood Collection Tubes (Sodium Heparin/Polyester Gel Samples) (Becton, Dickinson and Company, Franklin Lakes, NJ, USA), who received WT1 peptide pulsed DCs therapy before and after seven sessions of intradermal injections of DC vaccines every two weeks.

PBMCs were collected according to manufacturer’s instructions and cryopreserved using TC protector (DS pharma biomedical, Osaka, Japan) under a temperature condition of −80 to −180 °C.

### 2.4. Interferon (IFN)-γ ELISpot Assay

To assess the functional WT1-CTLs in PBMCs, IFN-γ producing cells were examined using the pre-coated Human IFN-γ ELISpot^PLUS^ Kit (HRP) (Mabtech, Nacka Strand, Sweden), according to manufacturer’s instructions. In brief, cryopreserved cells were thawed and the live cells were counted. Next, 1 × 10^6^ live cells/well were resuspended in AIM medium (Gibco, Gaithersburg, MD, USA) with 10% fetal bovine serum (BioWest, Nuaillé, France) in the presence of 10 μM of WT1 peptide or 10 μM of CMVpp65 peptide (QYDPVAALF, residue 341–349) (MBL, Medical & Biological Laboratories Co, Ltd, Nagoya, Japan). As a negative control, 10 μM HLA-A*24:02 human immunodeficiency virus (HIV) env (RYLRDQQLL, residue 584–592) (MBL, Nagoya, Japan) was used.

In some experiments, the ELISpot assay was performed using CD8^+^ T cells isolated from patient PBMCs after vaccination. The CD8^+^ T cells were isolated using microbeads conjugated to CD8 monoclonal antibodies (mAb) (Miltenyi Biotec, San Diego, CA, USA) and then cultured (3 × 10^5^ cells/well) as mentioned above in the presence of CD8^−^ cells pulsed with WT1 peptide as stimulator cells.

After 18–20 h of incubation at 37 °C and 5% CO_2_, the spots formed by IFN-γ-secreting cells were counted by an automated ELISpot reader (Autoimmun Diagnostika, Strassberg, Germany). Peptide specific spots were considered after deduction of spots of the negative control peptide from that of the WT1 peptide or the CMVpp65 peptide. Results were determined as the mean number of peptide specific spots per 1 × 10^6^ PBMCs from duplicated wells. The presence of WT1-CTLs was defined according to the following criteria: (1) at least 15 WT1-specific spots per 1 × 10^6^ PBMCs and (2) at least 1.5-fold more presence of WT1-specific spots than negative control peptide spots [11,12,13].

### 2.5. Tetramer Analysis

PBMCs were stained with phycoerythrin (PE)-conjugated WT1-modified peptide/HLA-A*24:02 tetramer (MBL, Nagoya, Japan) or PE-conjugated HIV envelope/HLA-A*24:02 tetramer (MBL, Nagoya, Japan) as a negative control, along with allophycocyanin-conjugated anti-human CD3 mAb (Biolegend, San Diego, CA, USA) and fluorescein isothiocyanate-conjugated anti-human CD8 mAb (Beckman Coulter, Miami, FL, USA). PBMCs were then analyzed by flow cytometry (FACSCalibur, BD Biosciences, San Jose, CA, USA). WT1-tetramer-positive CTLs were defined according to the following criteria: (1) comprising at least 0.02% in the CD3^+^CD8^+^ subset of 50,000–100,000 lymphocytes, and (2) forming a clustered and not diffused population.

Chudley and Britten *et al.* reported that the rate of detection in tetramer analysis was much lower with 10,000 CD8^+^ T cells than with 100,000 [14,15,16]. However, evaluation of 100,000 CD8^+^ T cells was very difficult because of the limited number of PBMCs available from most cancer patients. Therefore, cases with less than 10,000 available CD3^+^CD8^+^ cells were excluded.

### 2.6. Statistical Analyses

The statistical analysis was conducted using the R Software package, Version 3.0.2 (R foundation for Statistical Computing, Vienna, Austria). Pearson’s correlation coefficient was used to assess linearity. The correlation between results of the ELISpot assay and that of the Tetramer analysis was analyzed using the Spearman’s rank-correlation coefficient test. To determine the responses of WT1-CTLs between pre- and post-vaccination, a Wilcoxon signed rank test was applied. Differences were considered statistically significant at *p* < 0.05.

## 3. Results

### 3.1. Reproducibility of the ELISpot Assay

First, experiments were conducted to assess quantitative reproducibility of the ELISpot assay in three CMV-responder patients. 

To evaluate the repeatability of the ELISpot assay, each 15 wells of 1 × 10^6^ PBMCs stimulated with CMVpp65 peptide were examined. As shown in Table 1a, the CV ranged from 7.4% to 16.3%. Next, to evaluate the daily precision, each four wells of 1 × 10^6^ PBMCs with CMVpp65 peptide were analyzed on three different days. As shown in Table 1b, CV ranged from 5.0% to 17.3%.

**Table 1:** Precision of the ELISpot assay. The ELISpot assay was performed using peripheral blood mononuclear cells (PBMCs) from three cytomegalovirus (CMV)-responder patients. The number of spots per well are shown. (**a**) To evaluate the repeatability of the ELISpot assay, 1 × 10^6^ PBMCs were examined with CMVpp65 in each of 15 wells. (**b**) To evaluate the daily precision, 1 × 10^6^ PBMCs with CMVpp65 peptides were suspended in each of four wells and analyzed over three times on different days.

(a) Assay Reproducibility			
Sample	A	B	C
Mean	73.5	15.0	22.2
Median	73.0	14.0	24.0
SD	5.4	3.1	3.6
CV (%)	7.4	11.8	16.3
**(b) Daily Precision**			
Sample	A	B	C
Mean	77.4	24.6	20.1
Median	77.5	24.3	20.5
SD	3.9	4.3	3.1
CV (%)	5.0	17.3	15.7

### 3.2. Dilution Linearity

**Figure 1:**
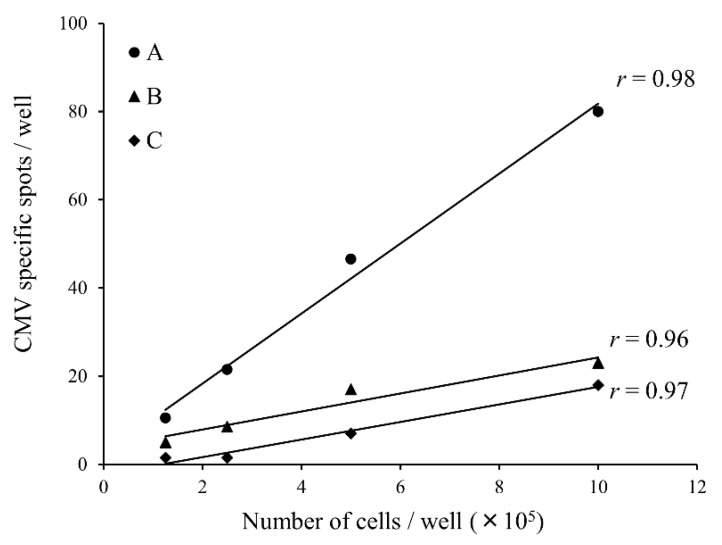
The linearity of ELISpot assay in sample dilution experiments. ELISpot assay was performed using peripheral blood mononuclear cells from three cytomegalovirus-responder patients with serial cell dilution (1.25 × 10^5^, 2.5 × 10^5^, 5.0 × 10^5^, 10.0 × 10^5^ cells/well) and CMVpp65 peptide. The mean of the CMV specific spots in duplicated wells was indicated in the graph. Pearson’s correlation coefficient (*r*) was 0.96–0.98.

To evaluate the dilution linearity, ELISpot assay was performed using PBMCs from three CMV-responder patients. Experiments were performed in serial cell dilution (1.25 × 10^5^, 2.5 × 10^5^, 5.0 × 10^5^, and 10.0 × 10^5^ cells/well) with CMVpp65 peptide. As shown in Figure 1, all three results showed high linearity (Pearson’s correlation coefficient *r* = 0.96–0.98).

### 3.3. Detection of WT1-Specific Immune Response by ELISpot Assay

**Figure 2:**
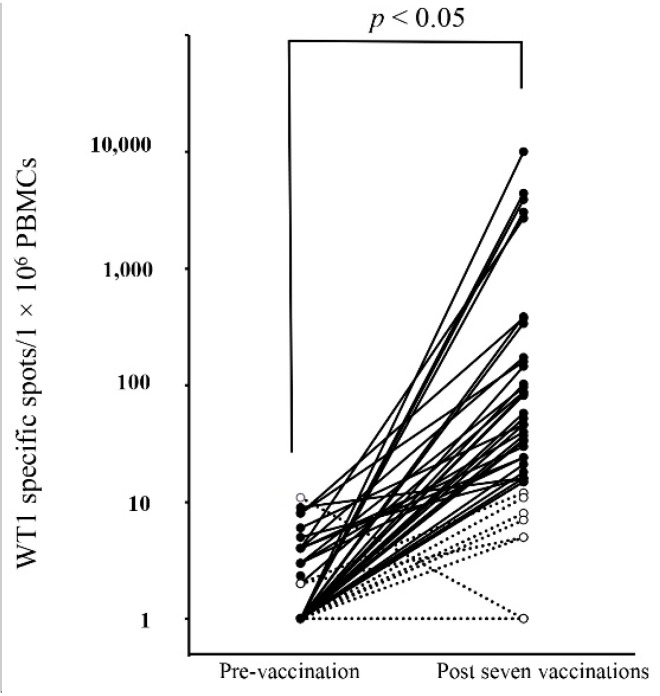
Assessment of Wilms’ tumor 1 (WT1)-specific immune response by ELISpot Assay. WT1-specific immune responses were analyzed both pre- and post-vaccination by ELISpot assay. Subjects were 46 patients who received WT1 peptide-pulsed dendritic cell therapy. Positive patients ●; Negative patients ○; WT1-specific cell responses were detected in 34 out of 46 patients (73.9%) after vaccination. Wilcoxon signed-rank test was *p* < 0.05. A representative positive case is shown in Figure 3.

As shown in Figure 2, WT1 specific responses analyzed by ELISpot assay were detected in 34 out of 46 cancer patients (73.9%) after seven pulsed DCs vaccinations of WT1 peptide. A Wilcoxon signed-rank test showed a statistically significant increase in WT1-specific T cell response from the pre- to post-vaccination (*p* < 0.05).

**Figure 3:**
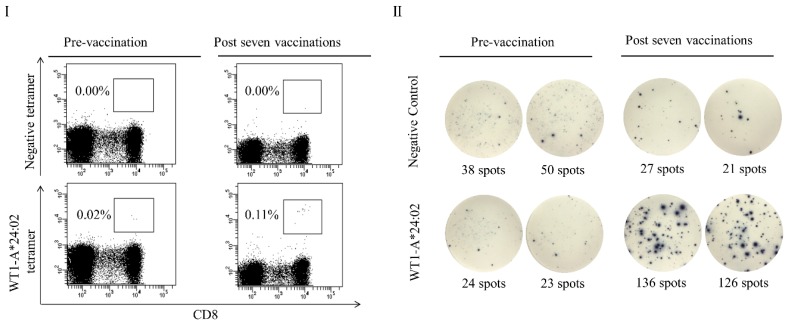
WT1 peptide-specific responses post-DC vaccination in a representative case. (**I**) The frequencies of CD8^+^ and Tetramer^+^ cells in the CD3^+^ population are shown. Numbers indicate the percentages of tetramer-positive cells within the CD8^+^ population. (**II**) ELISpot assays of PBMCs. WT1-specific IFN-γ secretion by PBMCs increased after vaccination.

### 3.4. IFN-γ Producing Cells in the PBMCs

To identify WT1 peptide-specific IFN-γ-producing cells among the CD8^+^ T cells, the ELISpot assay was performed using CD8^+^ T cells isolated from PBMCs of vaccinated patients. The CD8^+^ T cells (1 × 10^5^ cells/well) were cultured in the presence of CD8^−^ PBMCs pulsed with the WT1 peptide (2 × 10^5^ cells/well) at 37 °C for 30 min as stimulator cells.

**Figure 4:**
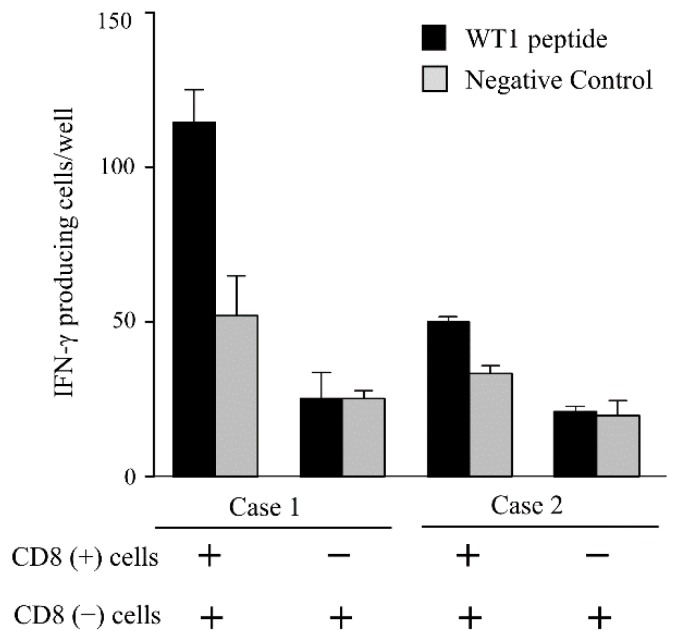
ELISpot assay results of two representative cases of CD8^+^ T cells isolated from PBMCs. The CD8^+^ cells (1 × 10^5^ cells/well) were cultured in the presence of CD8^−^ PBMCs pulsed with the WT1 peptide (2 × 10^5^ cells/well) as stimulator cells. Black and gray bars indicate the number of IFN-γ-producing cells per well stimulated by CD8^−^ cells pulsed with the WT1 peptide and with negative control peptide, respectively.

As shown in Figure 4, WT1-specific spots were detected only in the wells containing CD8^+^ cells in these cases, suggesting that WT1-specific IFN-γ-producing PBMCs were primarily CD8^+^.

### 3.5. Correlation between ELISpot Assay and Tetramer Analysis

**Figure 5:**
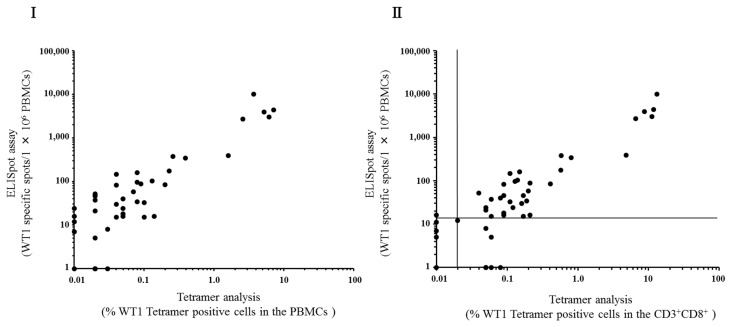
Correlation between the ELISpot assay and tetramer analysis. Subjects were 46 patients who were received WT1 peptide-pulsed DC therapy. Each tetramer data was rounded off to the second decimal place. The horizontal axis indicates the percentages of WT1 tetramer positive cells within PBMCs (**I**) or CD3^+^CD8^+^ population (**II**). The vertical axis indicates the number of WT1-specific IFN-γ secreting cells in 1 × 10^6^ PBMCs. Cut off line of each analysis was shown.

The ELISpot assay was compared with the tetramer analysis in 46 patients who were treated with WT1 peptide pulsed DC therapy. As shown in Figure 5(I), a significant positive correlation between the number of WT1 specific spots from 1 × 10^6^ PBMCs and the percentages of WT1 Tetramer^+^ cells in PBMCs was observed. The Spearman’s rank-correlation coefficient was 0.78. Figure 5(II) also showed a significant positive correlation between the number of WT1 specific spots from 1 × 10^6^ PBMCs and the percentages of WT1 Tetramer^+^ cells in CD3^+^CD8^+^ cells. The Spearman’s rank-correlation coefficient was 0.82, indicating a positive relationship between the ELISpot assay and the tetramer analysis. Percentage tetramers were rounded off to the second decimal point, resulting in very similar values between graph (I) and (II) for samples with low tetramer positive cells.

As shown in Table 2, WT1-specific immune responses were detected by both ELISpot and tetramer analyses in 33 out of 46 post-vaccination patients. Positive responses were detected in one patient by only ELISpot assay, and seven patients by only tetramer analysis. Accordingly, total positive results were detected in 41 out of 46 (89.1%) patients by either analysis.

**Table 2:** Cross tabulation of the findings by ELISpot and tetramer (CD3^+^CD8^+^) analyses from 46 cancer patients. The number of patients in each category is shown. WT1-specific immune responses were detected in 41 (89.1%) out of 46 patients post-vaccination by ELISpot and/or tetramer analysis.

-		ELISpot		
		+	−	Total
**Tetramer**	**+**	33	7	40
	**−**	1	5	6
	**Total**	34	12	46

## 4. Discussion

The evaluation of T-cell responses is very important to determine the effectiveness of vaccination-induced immune reaction. The ELISpot assay is considered as a unique method that allows the quantification of the actual cytokine secretory activity of individual cells among various approaches to evaluate T cell response [17,18].

In our facilities, the tetramer analysis has been performed to evaluate the efficacy of T cell responses in before and after seven-time application of WT1 peptide-pulsed DC vaccination. However, this test is limited as it detects only the frequency of epitope specific CTLs, all of which may not produce IFN-γ with antigen-specificity. On the other hand, the ELISpot assay can evaluate functional CTLs in response to stimulation by WT1 peptide. Therefore, using the IFN-γ ELISpot assay, we can assess not only the frequencies, but also the function of antigen-specific CTLs.

In the clinical setting, the validated methods should be used for immunological monitoring of the patients’ responses to treatment. Therefore, we assessed the reproducibility and linearity of the ELISpot assay. The results showed that there was reproducibility and daily precision as shown in Table 1. The dilution linearity results also showed a dose dependent response as shown in Figure 1. In the ELISpot assay, various concentrations (from 1 × 10^4^ to 1 × 10^6^ cells per well) and various types of cells have been used with many kinds of objective peptide [11,12,13,14,15]. However, the results of dilution linearity showed that when small numbers of cells were applied in a well, a very low number of antigen-specific T cells in the PBMCs may not be detected as Karlsson *et al.* reported [19]. Therefore, we decided to plate 1 × 10^6^ PBMCs per well for the ELISpot assay.

Next, we identified WT1-specific IFN-γ-producing populations of PBMCs using the ELISpot assay. As shown in Figure 4, IFN-γ-producing PBMCs in response to WT1 were indeed CD8^+^. Thus, we used PBMCs instead of bead-separated CD8^+^ cells in the ELISpot assay, which was easier and is more applicable to clinical settings.

We then assessed the correlation between the ELISpot assay and the tetramer analysis. A significant correlation was observed between WT1-specific spots from 1 × 10^6^ PBMCs and the percentages of WT1 tetramer positive cells in CD8^+^ cells, as shown in Figure 5(II). We also assessed the correlation between WT1-specific spots from 1 × 10^6^ PBMCs and the percentages of WT1 tetramer positive cells in the PBMCs in Figure 5(I). Although the percentages of WT1 tetramer positive cells in the PBMCs were lower than that of in the CD8^+^ population, there was a significant correlation between both analyses.

However, as shown in Table 2, some cases showed mismatched results between the ELISpot assay and the tetramer analysis. More specifically, seven cases (three cases of lung cancer and four of pancreatic cancer) were tetramer analysis positive and ELISpot assay negative. Tetramer analysis could detect T cells harboring T cell receptor (TCR) with sufficient affinity to bind to WT1-peptide. On the other hand, the IFN-γ ELISpot assay could detect the cells that secrete IFN-γ stimulated by WT1-peptide. Therefore, despite the WT1-specific T cells being phenotypically detected, they might have no functional release of IFN-γ in these seven cases.

Only one case of colorectal cancer was positively detected by ELISpot assay while not detected by tetramer analysis. In this case, the count of WT1-specific spots by ELISpot assay was 16, which was far smaller than other cases. However, the size of each spot was larger than that of the others. Therefore, a small number of WT1-specific CTLs that can produce a high amount of IFN-γ upon WT1 stimulation may exist. We propose that an important advantage of the ELISpot assay is its ability to detect small numbers of functional CTLs, which are otherwise undetectable via tetramer analysis in clinical assessment of cancer immunotherapy. However, the question remains as to whether these CTLs are sufficiently functional to convey anti-cancer effects *in vivo*. Thus, further investigation is warranted.

Because DC therapy is usually combined with chemotherapy and/or radiotherapy, a reduction of tumor size and a decrease of tumor markers do not always indicate the effect of DC therapy only [20,21,22]. However, the IFN-γ ELISpot assay and tetramer analysis would provide an indication of the efficacy of the DC therapy by detection of antigen specific CTLs [2,5,23,24]. Again, this is the first report to reveal the usefulness of the ELISpot assay in the context of DC vaccination anti-cancer therapy in comparison with tetramer analysis, which is widely used clinically to assess the effects of therapy.

In the scientific magazine *Science*, anti-cancer immunotherapy was chosen as the top scientific breakthrough of 2013 [25]. Immunotherapy will further develop as the fourth routine method of cancer treatment in the future. Within this context, the evaluation of monitoring methods assessing immunotherapy by an established clinical laboratory procedure will become increasingly important.

## 5. Conclusions

The ELISpot assay has reproducibility, linearity, and excellent correlation with the WT1 tetramer analysis. The validated ELISpot assay is useful to monitor the acquired immunity by DC vaccination targeting WT1.
